# Patients from Remote Health Centers Referred to Cayenne Emergency Department: A One-Year Observational Study

**DOI:** 10.4269/ajtmh.24-0705

**Published:** 2025-12-09

**Authors:** Marie Eva Miomandre, Rémi Mutricy, Florian Negrello, Félix Djossou, Cyril Rousseau, Antoine Adenis, Alexis Fremery

**Affiliations:** ^1^ Emergency Department, Cayenne General Hospital, Cayenne, French Guiana;; ^2^ Emergency Department, University Hospital of Martinique, Fort de France, Martinique;; ^3^ Tropical and Infectious Diseases Department, Cayenne General Hospital, Cayenne, French Guiana;; ^4^ French Guiana University, Cayenne, French Guiana;; ^5^ Remote Health Center Department, Cayenne General Hospital, Cayenne, French Guiana;; ^6^ CIC INSERM1424, Cayenne General Hospital, Cayenne, French Guiana

## Abstract

French Guiana has developed a health organization to respond to its geographical situation. Remote health centers provide primary and emergency care in isolated areas. The limited technical facilities at the remote health centers result in a significant number of patient transfers to the Cayenne emergency department (ED). The objective of this study was to describe the epidemiology and management of patients transferred to Cayenne ED. A retrospective observational study was conducted from January 1 to December 31, 2019, and it included all patients transferred from remote health centers to Cayenne ED. All sociodemographic, prehospital, hospital, and referral data were collected; 842 patients were transferred by remote health centers to the Cayenne ED. The male/female ratio was 1.27, with an age of 36 (±23) years old. The two main modes of transportation used were helicopter (36%) and plane (22%). The most frequent reasons for transfer were trauma (28%), digestive (9%), respiratory (9%), and infectious (8%) conditions. Patients were hospitalized in 71% of cases. Among patients who were not hospitalized, 7% did not require further examination or specialist advice in the ED. Our work underlines the important use of airborne resources, particularly medical ones; they were initially intended for the management of vital emergencies, but they are also used for nonurgent situations. The geography and road access in French Guiana make alternative means of transport difficult. Our work identifies a number of areas for optimizing care to decrease the number of transfers: improving biomedical equipment, improving imaging equipment, and use of telemedicine.

## INTRODUCTION

French Guiana (FG) is a South American territory covered by tropical forest, and it has a poorly developed road network. Its large surface area (83,000 km^2^) contrasts with its low population density (3.3 inhabitants/km^2^), with 80% of the population concentrated on the coast.^1^^,^^2^ Population growth continues at a steady pace of +2.5% per year, mainly in the remote cities of western FG: Grand Santi (+8.2%), Papaïchton (+7.1%), and Maripasoula (+6.2%). The population of FG is very disadvantaged, with high levels of social inequalities, particularly in terms of health care access.^3^ In 2017, 53% of Guianese were living below the national poverty threshold (<1,010 euros per month).^4^

Despite numerous efforts to develop the health care network, access to health care remains highly heterogeneous. The three largest coastal towns have hospitals with emergency departments (EDs), laboratories, and operating rooms: one in Cayenne city, one in Saint Laurent du Maroni, and one in Kourou.^5^ Health care in remote areas is provided by remote health centers (RHCs). There are 17 of them in FG; eight of the centers have a 24-hour medical presence, and nine are nursing stations with discontinuous presence.^6^ Six of these centers, covering an FG population area of 11%, are accessible only by river or air. To compensate for this lack of access, the emergency medical service (EMS) uses helicopters to reach isolated populations.^7^ The average estimated round-trip time to access the ED by air is approximately 3 hours.

In 2014, the RHCs provided 1,900 transfers to hospitals and 179,000 consultations carried out at five main centers: Maripasoula (18%), Grand Santi (15%), Saint Georges (13%), Apatou (11%), Papaïchton (8%), and Camopi (7%).^8^ In 2007, the majority of activity was devoted to pediatrics (47%).^6^ The equatorial climate and rich biodiversity are also conducive to infectious pathologies and envenomations.^9^^–^^11^ Trauma is also very common, as are life-threatening emergencies.^12^ The care provided by RHCs is adapted to the local population and their specific needs.^9^^,^^13^ The diversity of the RHC’s mission is complicated by limited technical facilities; the only imaging available is ultrasound, biological samples are sent to laboratories within 24–72 hours, and access to specialists is limited, justifying frequent transfers to coastal hospital EDs.^12^

The primary objective of this study is to describe the epidemiology and management of patients transferred from French Guianese RHCs to the Cayenne ED.

## MATERIALS AND METHODS

### Study design.

We conducted a retrospective, descriptive, monocentric study of patients transferred by RHCs of FG to the ED of the Cayenne Hospital from January 1, 2019 to December 31, 2019.

### Study population.

All patients admitted to the Cayenne Hospital (ED) transferred from an RHC were included in the study. Exclusion criteria for data collection were patients not referred by an RHC, direct hospitalization in a specialized department, patients who left the hospital before the end of emergency care, computerized records not found or duplicates, and medical records created by mistake. We chose not to include gynecological emergency records because they are not part of the general ED. We did, however, analyze data from pediatric patients (younger than 15 years old) managed within the general ED.

### Data sources and variables.

We extracted the ED records using the postal code corresponding to patients from isolated areas. We crosschecked these data with the hospitalization summary using data on patients from isolated towns hospitalized after a visit to the ED. From these spreadsheets, we searched the computerized records of all software used for the medical record of Cayenne’s ED (the DMU™ software, Atos, Bezons, France) to specify the patient’s origin (RHC, other health professional, or nonreferred patient) and extract the necessary medical data. We consulted CORA™ computerized hospitalization reports (Maincare Corporate, Canejan, France) to collect the examinations performed during the management and the final diagnosis in case of hospitalization. Medical laboratory software SRI™ (AGFA Healthcare, Mortsel, Belgium) and imaging software Xplore™ (EDL, Lyon, France) were consulted to confirm the performance of complementary examinations. Demographic variables, such as place of birth and social security coverage, were extracted from Hextant™ (Dedalus France, Le Plessis-Robinson, France). Sociodemographic variables, data on prehospital care and management in the ED, diagnoses and patient referral, length of stay in the ED, and total length of hospital stay were collected. All data were transcribed anonymously into an Excel™ spreadsheet (Microsoft Corp., Redmond, WA) before statistical analysis. No data were directly identifiable; each patient was assigned a number in order of inclusion.

## STATISTICAL ANALYSES

Categorical variables were described in terms of numbers, percentages, and CIs. Continuous variables were described in terms of means and SDs or medians and interquartile ranges depending on the normality of the distribution of the variable studied. Comparisons of stay characteristics by origin, age (adult versus child), and hospitalization or not were subjected to the Fisher exact test and the Student’s *t*-test. Results were expressed as odd ratios and mean differences (µ) with CIs and *P*-values. Figures were produced using R™ software v. 4.1.0 (R Foundation, Vienna, Austria) and Excel software, and tables were produced using R software v. 4.1.0 with an α threshold set at 5%.

## RESULTS

### General population.

**Figure 1.**
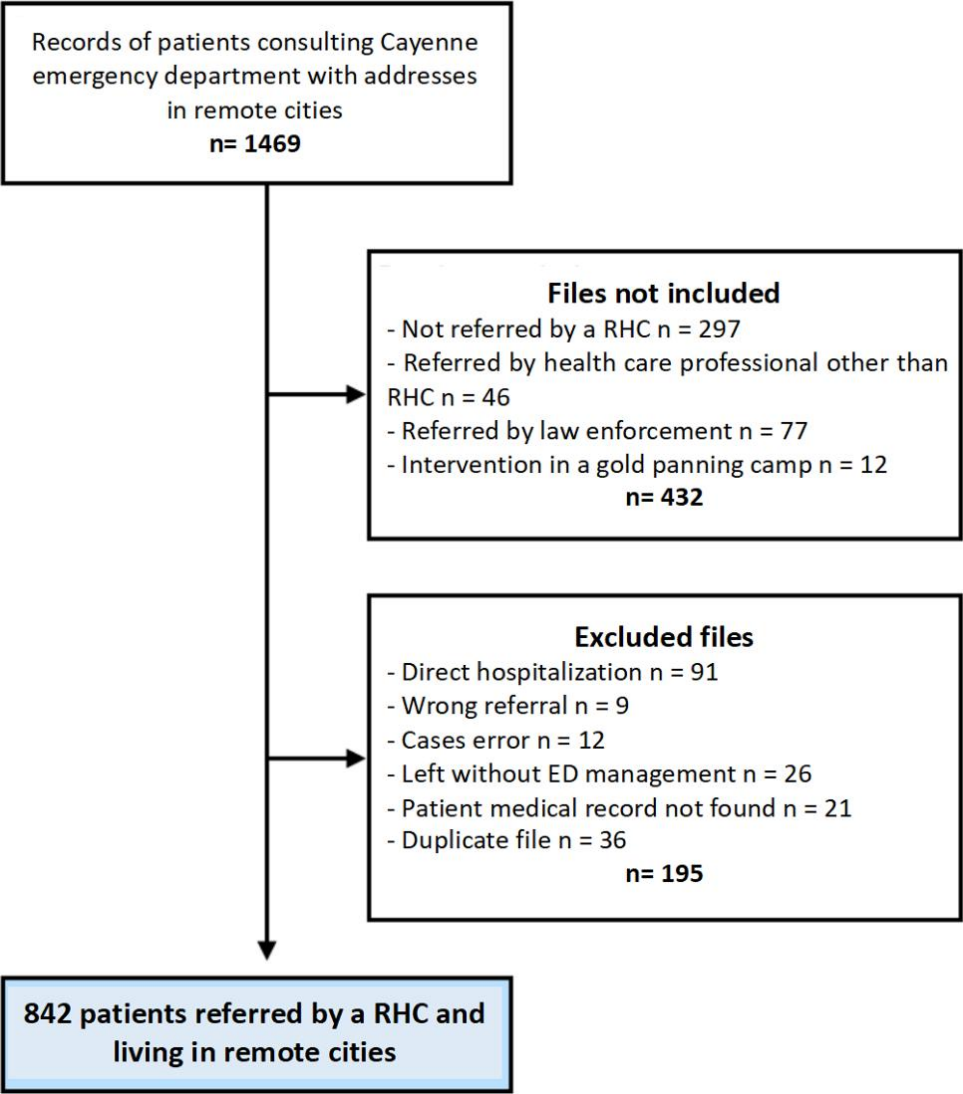
Study flowchart of patient record selection from the Cayenne emergency department (ED). RHC = remote health center.

**Figure 2.**
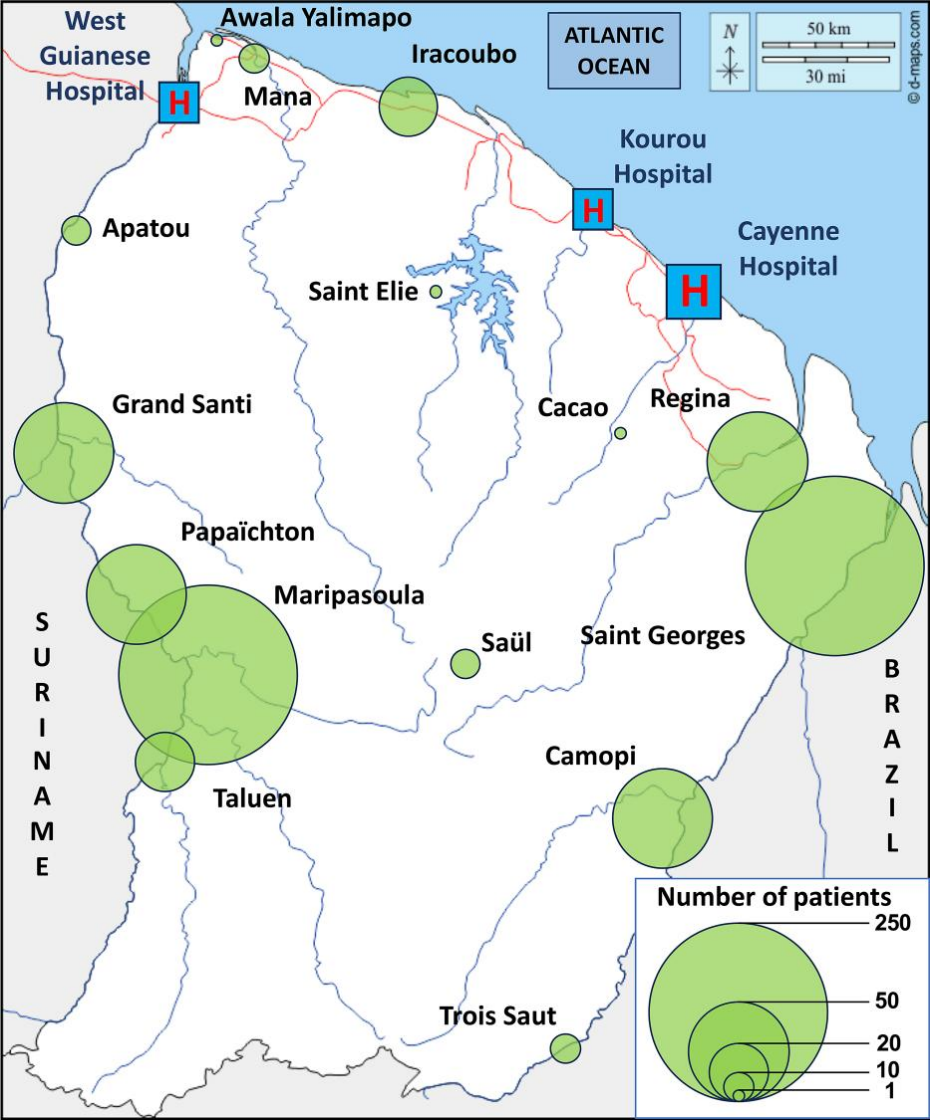
Proportional geographic representation of patient origins of patients referred by remote health centers to the Cayenne emergency department in 2019.

From January 1 to December 31, 2019, 1,469 patients from isolated areas were registered in the ED records. Of these, 627 files that did not meet the inclusion criteria were not included in the study. The study flowchart is presented in Figure 1. The total population (*n* = 842) included 650 (77%) adults (older than 15 years old) and 192 (23%) children (younger than 15 years old). The sex ratio (male/female) of the population was 1.27. The mean age was 36 (±23) years old. More than half of the transferred population was between 26 and 65 years old (*n* = 453). Supplemental Appendix 1 shows the age distribution of the population. The characteristics of our study population are presented in Table 1. The majority of the population was of French Guianese (*n* = 439, 53%) and Brazilian (*n* = 251, 30%) origin. The native language item had a large amount of missing data (*n* = 276, 33%), but the most frequent answer was Brazilian Portuguese (*n* = 254, 44%). The RHCs of Saint Georges and Maripasoula transferred the majority of the population (32% each). The patient origins by RHCs are shown in Figure 2.

**Table 1:** Description of the population transferred to the Cayenne emergency department

Variables	Adult (*n =* 650)*	Children (*n =* 192)*	Total (*N =* 842)*
Sex (male)	371 (57%)	103 (54%)	474 (56%)
Origin			
Brazil	242 (38%)	9 (4.8%)	251 (30%)
Mainland France	43 (6.7%)	5 (2.7%)	48 (5.8%)
French Guiana	271 (42%)	168 (89%)	439 (53%)
Suriname	60 (9.3%)	5 (2.7%)	65 (7.8%)
Other	26 (4.0%)	1 (0.5%)	27 (3.3%)
City			
Maripasoula	211 (32%)	56 (29%)	267 (32%)
Saint Georges	205 (32%)	61 (32%)	266 (32%)
Camopi	50 (7.7%)	14 (7.3%)	64 (7.6%)
Grand Santi	37 (5.7%)	19 (9.9%)	56 (6.7%)
Other	147 (23%)	42 (22%)	189 (22%)
Social security			
Yes	350 (55%)	149 (78%)	499 (60%)
SMC	70 (11%)	35 (18%)	105 (13%)
Native language			
Native American^†^	70 (15%)	25 (28%)	95 (17%)
Brazilian Portuguese	230 (48%)	20 (22%)	250 (44%)
French	75 (16%)	7 (7.8%)	82 (14%)
Dutch	13 (2.7%)	2 (2.2%)	15 (2.7%)
Taki taki	72 (15%)	36 (40%)	108 (19%)
Other	16 (3.4%)	0 (0%)	16 (2.8%)

SMC = state medical care.

* *n* (%) or mean (SD).

^†^ Native American languages include Kali’na, Palikour, Arawak, Wayana, Wayampi, and Emerillon.

### Prehospital care.

**Figure 3.**
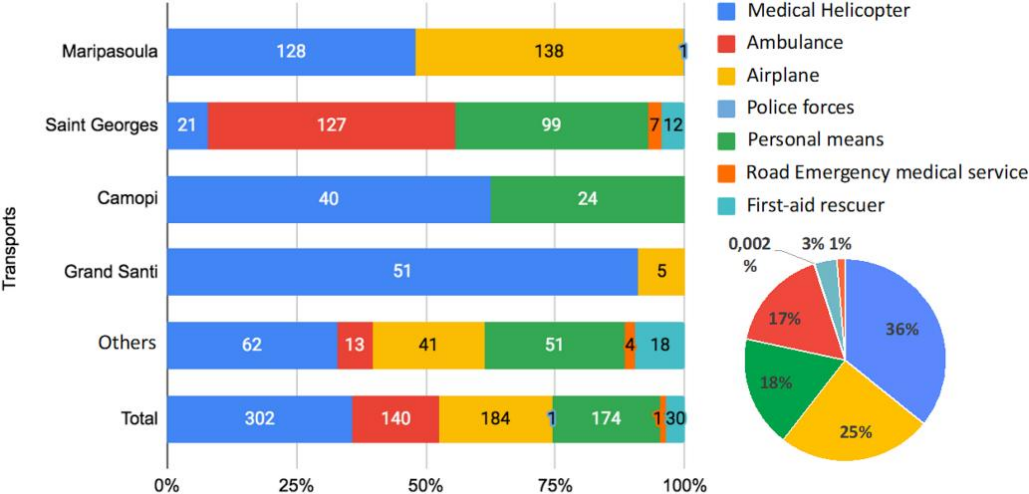
Distribution of transports used by remote health centers.

Transport methods used for transfers are shown in Figure 3. Helicopters were the most popular means of transport (*n* = 301, 36%). They were most frequently used for remote towns with no road access: Maripasoula (48%), Grand Santi (91%), and Camopi (62%). Traumatology was the most frequent reason for transfer (*n* = 237, 29%). Among trauma diagnoses (Supplemental Appendix 2), fractures were the most frequent (*n* = 99, 41%). However, more than half of all diagnoses were minor traumas, such as contusions (*n* = 43, 18%), wounds (*n* = 41, 17%), and sprains (*n* = 23, 9%). Other frequent reasons were digestive (*n* = 79, 10%), respiratory (*n* = 75, 9%), and infectious diseases (*n* = 66, 8%).

Potential surgical emergencies included appendicitis (*n* = 13, 16%) and cholecystitis (*n* = 10, 12%). The primary reason for respiratory referral was the diagnosis of pneumonia (*n* = 42, 55%) followed by bronchiolitis (*n* = 13, 17%). Infectious diagnoses were varied: HIV (*n* = 7, 10%), tuberculosis (*n* = 4, 5%), and toxoplasmosis (*n* = 3, 4%). Cardiology accounted for 63 (7%) transfers, including 33% (*n* = 21) for cardiac decompensation and 11% (*n* = 7) for acute coronary syndromes. Neurological reasons (*n* = 51, 6%), less frequent in our cohort, were related to ischemic and hemorrhagic stroke diagnoses (*n* = 23, 47%).

### Emergency department management.

The management of patients in the ED according to whether the patient was hospitalized or not is detailed in Table 2. Hospitalizations accounted for 71% (*n* = 597) of the cohort. The Clinical Classification of Emergency Patients (CCMU) (Supplemental Appendix 3) is commonly used in France to assess patient severity on arrival at the ED. The majority of patients had a CCMU between two (*n* = 338, 41%) and three (*n* = 321, 39%). For hospitalized patients, laboratory tests were managed for 498 patients (83%), imaging studies were managed for 423 patients (71%), and specialist evaluation was requested for 90% (*n* = 536) of hospitalized patients. Among the nonhospitalized group (*n* = 243, 29%), 40% (*n* = 98) requested a laboratory test, 56% (*n* = 136) had a specialist evaluation, and 64% (*n* = 98) of patients received an X-ray. Table 3 presents the hospitalization rate relative to the chief complaints and diagnosis. Infectiology and dermatology were the main medical chief complaints (*n* = 186) with an hospitalization rate of 86%, whereas trauma (*n* = 236) presented only a hospitalization rate of 59.3%. Odds ratios present the risk to be hospitalization relative to all other complaints.

**Table 2:** Emergency management depending on whether the patient was hospitalized

Characteristics	Nonhospitalized (*n =* 243)*	Hospitalized (*n =* 597)*	Total (*N =* 840)*	Effect Size^†^	*P*-Value
Adult	203 (84%)	445 (75%)	648 (77%)	OR = 0.58 [0.38–0.86]	**0.005**
Transport					
Medical helicopter	22 (9.1%)	277 (46%)	299 (36%)	OR = 8.7 [5.4–15]	**<0.001**
Ambulance	47 (19%)	93 (16%)	140 (17%)	OR = 0.77 [0.52–1.2]	0.19
Plane	63 (26%)	121 (20%)	184 (22%)	OR = 0.73 [0.51–1.1]	0.081
Personal means	99 (41%)	75 (13%)	174 (21%)	OR = 0.21 [0.14–0.30]	**<0.001**
Roadside EMS	2 (0.8%)	9 (1.5%)	11 (1.3%)	OR = 1.8 [0.38–18]	0.53
First-aid rescuer	10 (4.1%)	20 (3.4%)	30 (3.6%)	OR = 0.81 [0.36–2.0]	0.68
Biology	98 (40%)	498 (83%)	596 (71%)	OR = 7.4 [5.2–11]	**<0.001**
Imagery	154 (63%)	423 (71%)	577 (69%)	OR = 1.4 [1.0–2.0]	**0.033**
Ultrasonography	13 (8.5%)	39 (9.2%)	52 (9.0%)	OR = 1.1 [0.55–2.3]	0.87
MRI	1 (0.7%)	14 (3.3%)	15 (2.6%)	OR = 5.2 [0.78–222]	0.13
X-ray	98 (64%)	185 (44%)	283 (49%)	OR = 0.44 [0.29–0.65]	**<0.001**
CT scan	41 (27%)	184 (44%)	225 (39%)	OR = 2.1 [1.4–3.3]	**<0.001**
Specialist evaluation	136 (56%)	536 (90%)	672 (80%)	OR = 6.9 [4.7–10]	**<0.001**
Additional examinations					
Endoscopy	5 (2%)	20 (3%)	25 (5%)	OR = 0.00 [0.00–6.1]	0.56
Coronarography	–	5 (0.8%)	5 (0.8%)	OR = Inf [0.17–Inf]	0.56
Treatment	181 (74%)	530 (89%)	711 (85%)	OR = 2.7 [1.8–4.1]	**<0.001**
Analgesia	69 (38%)	40 (7.5%)	109 (15%)	OR = 0.13 [0.08–0.21]	**<0.001**
Antibiotics	25 (14%)	146 (28%)	171 (24%)	OR = 2.4 [1.5–3.9]	**<0.001**
Surgery	–	125 (24%)	125 (18%)	OR = Inf [15–Inf]	**<0.001**
Medical management	60 (33%)	207 (39%)	267 (38%)	OR = 1.3 [0.89–1.9]	0.18
Orthopedic treatment	27 (15%)	12 (2.3%)	39 (5.5%)	OR = 0.13 [0.06–0.28]	**<0.001**
ED length of stay (hours)	9 (10)	15 (17)	13 (15)	µ = 5.9 [4.1–7.8]	**<0.001**

CT = computed tomography; ED = emergency department; EMS = emergency medical service; Inf = infinity; MRI = magnetic resonance imaging; OR = odd ratio. Results are expressed as mean differences (µ) and ORs. When µ is >0, this means that the value is on average higher in children (and vice versa if µ <0). When the OR is >1, this means that the factor is more frequent in adults (and vice versa if OR <1). Values in bold indicate statistically significant *p*-values (*p* < 0.05).

* *n* (%) or mean (SD).

^†^ Mean differences or ORs.

**Table 3:** Hospitalization rate and ratio by presenting complaints specialty

Complaints Specialty	*N*	Hospitalization Rate, %	OR* (95% CI)
Toxicology	23	91.3	4.40 [1.28–27.67]
Metabolic/endocrinology	22	90.9	4.19 [1.21–26.36]
Forensic	10	90.0	3.71 [0.69–68.66]
Infectiology/dermatology	186	86.0	3.06 [1.99–4.87]
Hematology	12	83.3	2.06 [0.54–13.45]
Neurology	54	77.8	1.46 [0.78–2.95]
Cardiology	71	73.2	1.13 [0.66–2.00]
Digestive/urology	93	72.0	1.06 [0.66–1.74]
Pneumology	23	69.6	0.93 [0.39–2.45]
Oncology	6	66.7	0.81 [0.16–5.91]
Psychiatry	54	61.1	0.62 [0.35–1.11]
Rheumatology	10	60.0	0.61 [0.17–2.40]
Traumatology	236	59.3	0.47 [0.34–0.65]
Ophthalmology	8	50.0	0.40 [0.09–1.72]
Otolaryngology	34	41.2	0.27 [0.13–0.54]

OR = odds ratio.

* ORs are presented as complaint versus all other complaints.

**Figure 4.**
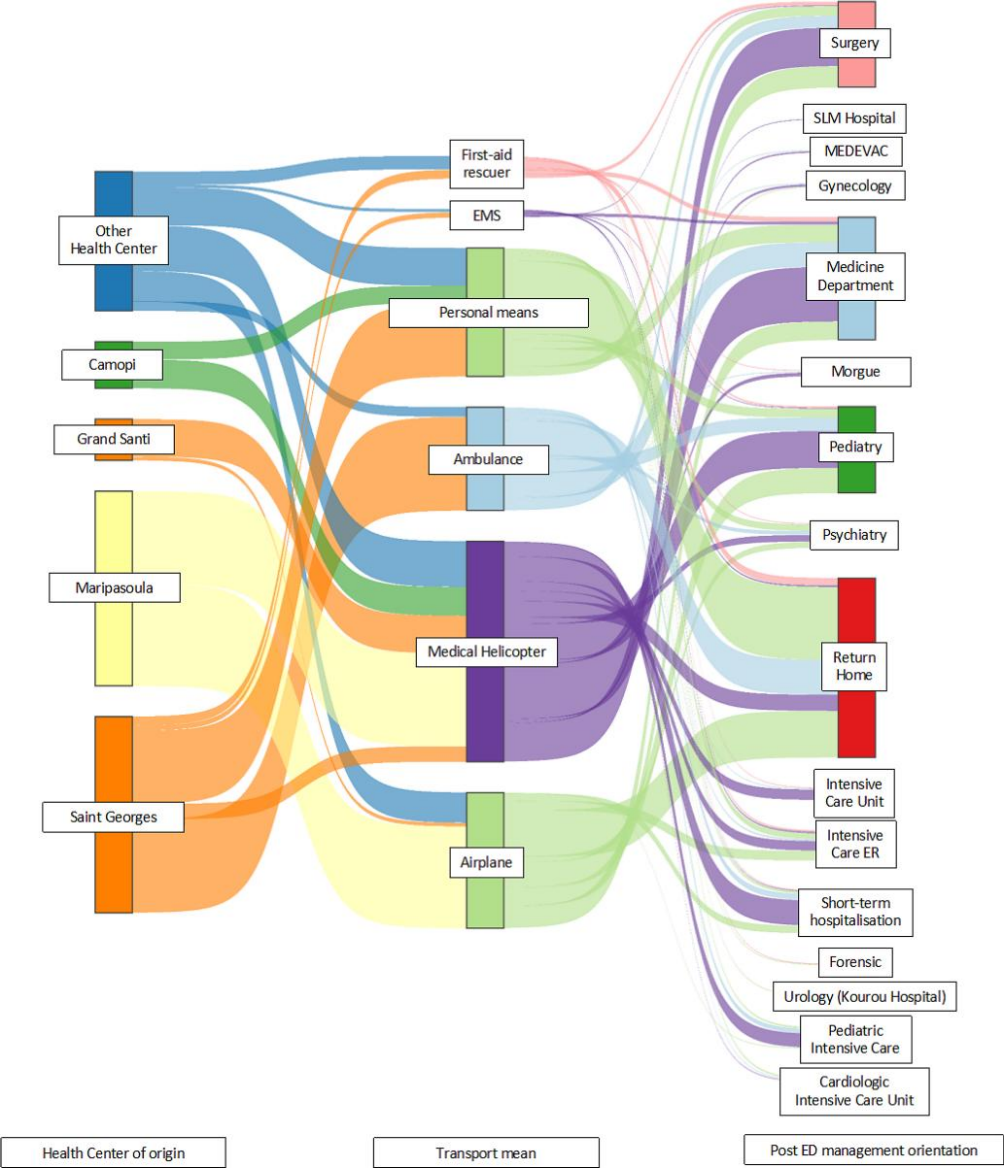
Sankey diagram representing the overall flow of the study population. ED = emergency department; EMS = emergency medical service; ER = emergency room; MEDEVAC = medical evacuation; SLM = Saint-Laurent-du-Maroni.

The Sankey diagram in Figure 4 shows the overall flow of patients in our study. It represents the patient journey in three stages: from the health center to the means of transport and then, to referral from the ED. In Figure 4, the width of the arrow is proportional to the patient flow. This gives an overview of the actual population referred by the health centers and managed in the Cayenne ED and their eventual outcome.

## DISCUSSION

This study describes the epidemiology and management of patients referred from FG health centers to the Cayenne ED in 2019. We counted 842 patients, representing 3% of the official population of the remote cities and 1.7% of the annual consultations at the Cayenne ED. The population referred was young and male. The most frequently used means of transport were EMS helicopter (36%), plane (22%), and personal means (21%). Saint Georges and Maripasoula accounted for the majority of transfers: the former likely because of its easy road access to Cayenne and the latter because it has the largest population. The most frequent reasons for transfer were trauma (29%), digestive (9%), respiratory (9%), and infectious diseases (8%). Many patients were able to return home (29%). Patients were hospitalized in two thirds of cases, with a mean length of stay in the ED of 13 hours.

Brazilian Portuguese was the language spoken in 39% of cases as there is a population originating from this neighboring country in part because of the very active gold-panning activity in the surrounding area.^14^^,^^15^ In our study, a quarter of patients transferred from Maripasoula (*n* = 68) had no social security coverage, whereas this rate reached 29% in Grand Santi. In 70% of cases, they came from Brazil, and in 6% of cases, they came from FG. The populations of the remote cities are characterized as precarious.^4^^–^^6^^,^^16^ The geographical isolation of the cities, their disadvantaged social situation, the lack of social workers, and direct discrimination are all factors favoring social insecurity.^17^ Among the priorities of the 2018–2027 Regional Health Project led by the Regional Health Agency are improving access to social rights related to health coverage and addressing the issue of care refusal. This will involve, in particular, the creation of a specific health access pathway and the installation of social protection delegates in the most isolated places.^7^

There was heavy use of medical helicopters: 301 transfers (36%) or 1,000 flight hours, corresponding to a theoretical cost of around 2 million euros. This represents half of the annual heliborne transports carried out by the EMS in FG.^7^ Another point to emphasize is the difficulty of accessing air tickets on commercial flights to transfer patients to the ED (availability, weather conditions, etc.). As the hospital does not have its own airplanes, this impacts the transfer of midurgent patients. In some cases, delays in transfer reduce patient outcomes, which may ultimately require deploying a helicopter to provide appropriate care. Having its own airplane is one of the planned projects for the new university hospital of FG in 2026.

Telemedicine was introduced in FG in the 2000s in an attempt to overcome the shortage of specialists in isolated areas.^18^ This was made possible by Cayenne General Hospital’s collaboration with MEDEcine Spatiale and the Centre National d’Etudes Spatiales, which created a portable telemedicine station linked to the satellite network. Initially created to meet the needs of dermatology, infectiology, and cardiology specialties, it now applies to all specialties.^19^ A study assessing the quality of the teledermatology service in French Guianese health centers showed that of 204 notifications recorded over 18 months (2015–2016), 8% of health center patients had to be evacuated. The study suggests that 92% of face-to-face specialist consultations could have been avoided as well as the resulting cost of transport and hospitalization.^20^ Telemedicine has significantly improved access to health care in some of the most remote regions of the world, particularly in the Amazon.^21^^–^^23^ In FG, it should serve as a major lever to enhance access to care while reducing the need for patient transfers.

In our work, trauma accounted for a third of emergency transfers and 41% of returns home. By equipping health centers with X-ray equipment and a high-speed network providing high-quality coverage for telemedicine, we can assume that for trauma, 40% of patients could be cared for in health centers without being transferred. One of the objectives is to integrate telemedicine in health centers as a decision support tool for medical regulation (emergency services, health care establishments, health centers, and border establishments). This functionality would help reduce the number of medical evacuations. Local social pressure, multilingualism that complicates obtaining reliable information, and sometimes, the lack of experience in the emergency may cause the EMS to transfer excessively.^19^

One way of improving emergency practices in health centers could be to use in situ simulation.^24^ This original strategy enables us to analyze the entire organization of care, with the advantage of immersion in a world close to reality. Trainers from Cayenne ED could use a mobile unit to travel to the health centers to train the teams.^25^ Apart from the acquisition of technical skills, this would reinforce team cohesion and the functioning of group practice. The cooperation project involving the insertion of EMS correspondents has been tested since May 2022.^12^ Health centers are the main players in the care of people in isolated areas in FG. French Guiana is part of a vast health reorganization project, with the creation of a multisite regional university hospital in 2025.^7^^,^^26^ In this context, it seems essential to strengthen the role of the health centers, which are facing explosive demographic pressure, and to label them as local hospitals for the main centers of Maripasoula, Saint Georges, and Grand Santi. To make the health centers the fourth pillar of the university hospital and decrease transfers, it will be necessary to modernize equipment, install imaging and laboratory resources, and create short-term hospitalization medical units. These measures will require the close coordination of all local players: the state, local authorities, health care professionals, and operators.

## CONCLUSION

The preventive and curative work carried out by the RHCs is combined with the management of vital and relative emergencies. French Guiana’s geography makes transport alternatives difficult. Strengthening human resources and ensuring their long-term sustainability are essential if preventive care and the monitoring of chronic pathologies are to be considered on a long-term basis. Continuing the use of telemedicine systems, installing X-ray equipment, and setting up laboratories will reduce the number of emergency transfers from RHCs to EDs.

- Total initial records: 1,469 patients with addresses in remote cities (Figure 1).
- First exclusion step: 432 files removed, including those not referred by an RHC (297), those referred by other health care professionals (46), those referred by law enforcement (77), and those involving interventions in gold-panning camps (12) (Figure 1).
- Second exclusion step: 195 files removed, including direct hospitalizations (91), wrong referrals (9), case errors (12), patients who left before ED management (26), missing medical records (21), and duplicate files (36) (Figure 1).
- Final dataset: 842 patients referred by an RHC and living in remote cities (Figure 1).
- Each remote city is represented by a green circle in Figure 2, where the size of the circle is proportional to the number of referred patients.
- Larger circles appear in Maripasoula, Grand Santi, and Saint Georges in Figure 2, indicating a high number of patients from these areas.
- Other contributing towns include Apatou, Papaïchton, Camopi, Saül, Trois Saut, Regina, Mana, Iracoubo, and Awala Yalimapo (Figure 2).
- Four hospitals are marked with red “H” symbols in blue boxes in Figure 2: West Guianese Hospital, Kourou Hospital, Cayenne Hospital, and another hospital in the northwest.
- A scale legend in the lower right of Figure 2 shows circle sizes corresponding to 1, 10, 20, 50, and 250 patients.
- Maripasoula: 128 medical helicopter transfers, 138 transfers by ambulance, and 1 transfer by plane (Figure 3).
- Saint Georges: 21 medical helicopter transfers, 127 transfers by ambulance, 99 transfers by plane, and 7 transfers by police forces (Figure 3).
- Camopi: 40 medical helicopter transfers and 24 transfers by personal means (Figure 3).
- Grand Santi: 51 medical helicopter transfers and 5 transfers by personal means (Figure 3).
- Other localities: 62 medical helicopter transfers, 13 transfers by ambulance, 41 transfers by plane, 5 transfers by police forces, and 18 transfers by personal means. Overall totals: 302 medical helicopter transfers (36%), 184 transfers by plane (25%), 140 transfers by ambulance (17%), 47 transfers by personal means (18%), 17 transfers by police forces (3%), and less than 1% transfers by other means. A pie chart visually represents these proportions is in Figure 3.
- Origin centers: Camopi, Grand Santi, Maripasoula, Saint Georges, and other centers (Figure 4).
- Transport methods: rescue workers, EMS, personal means, ambulance, medical helicopter, and airplane (Figure 4).
- Final orientations: surgery, SLM hospital, medical evacuation, gynecology, internal medicine, morgue, general medicine, psychiatry, discharge home, adult and pediatric intensive care, pediatric emergency, short-stay hospitalization, cardiology intensive care, and transfer to other facilities. The thickness of each flow line in Figure 4 represents the relative proportion of patients for each origin–transport–destination combination.

## Supplemental Materials

10.4269/ajtmh.24-0705 Supplemental Materials
