# Supplementary material for: Patients from Remote Health Centers Referred to Cayenne Emergency Department: A One-Year Observational Study
Source: Am J Trop Med Hyg. 2025 Dec 9;114(2):384–91. doi: 10.4269/ajtmh.24-0705 (PMC12874922; doi:10.4269/ajtmh.24-0705)
Supplement: Supplemental Materials [file tpmd240705.SD1.pdf]

APPENDICES – Online only

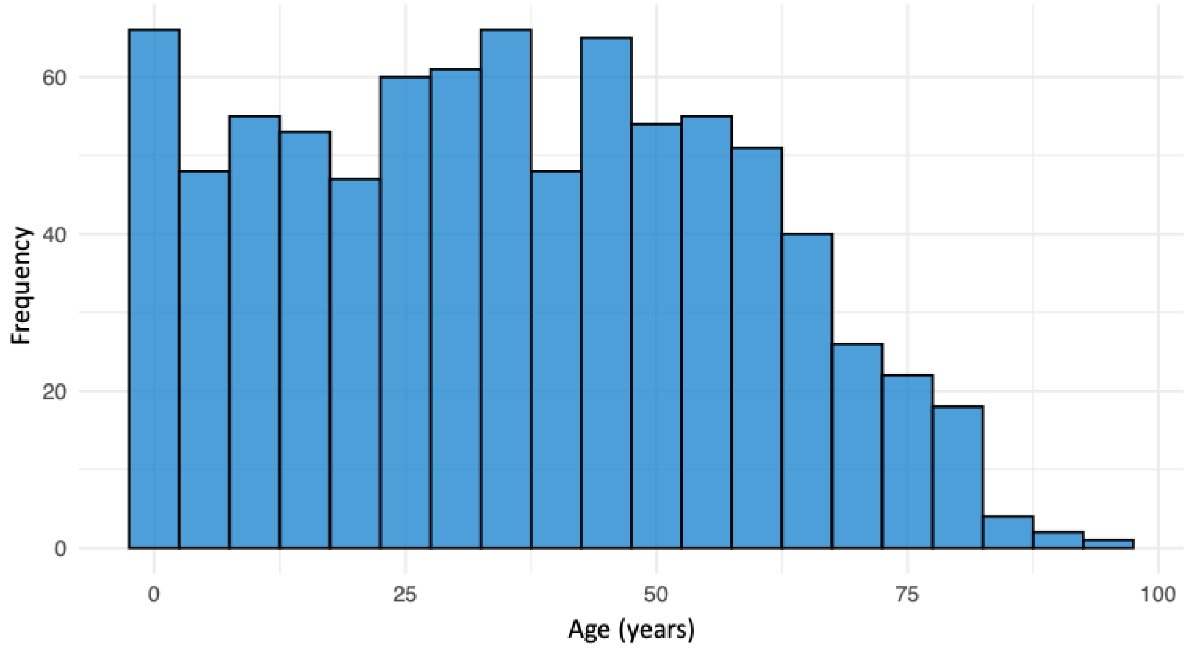

Appendix 1: Age distribution of the RHC population referred to the emergency department.

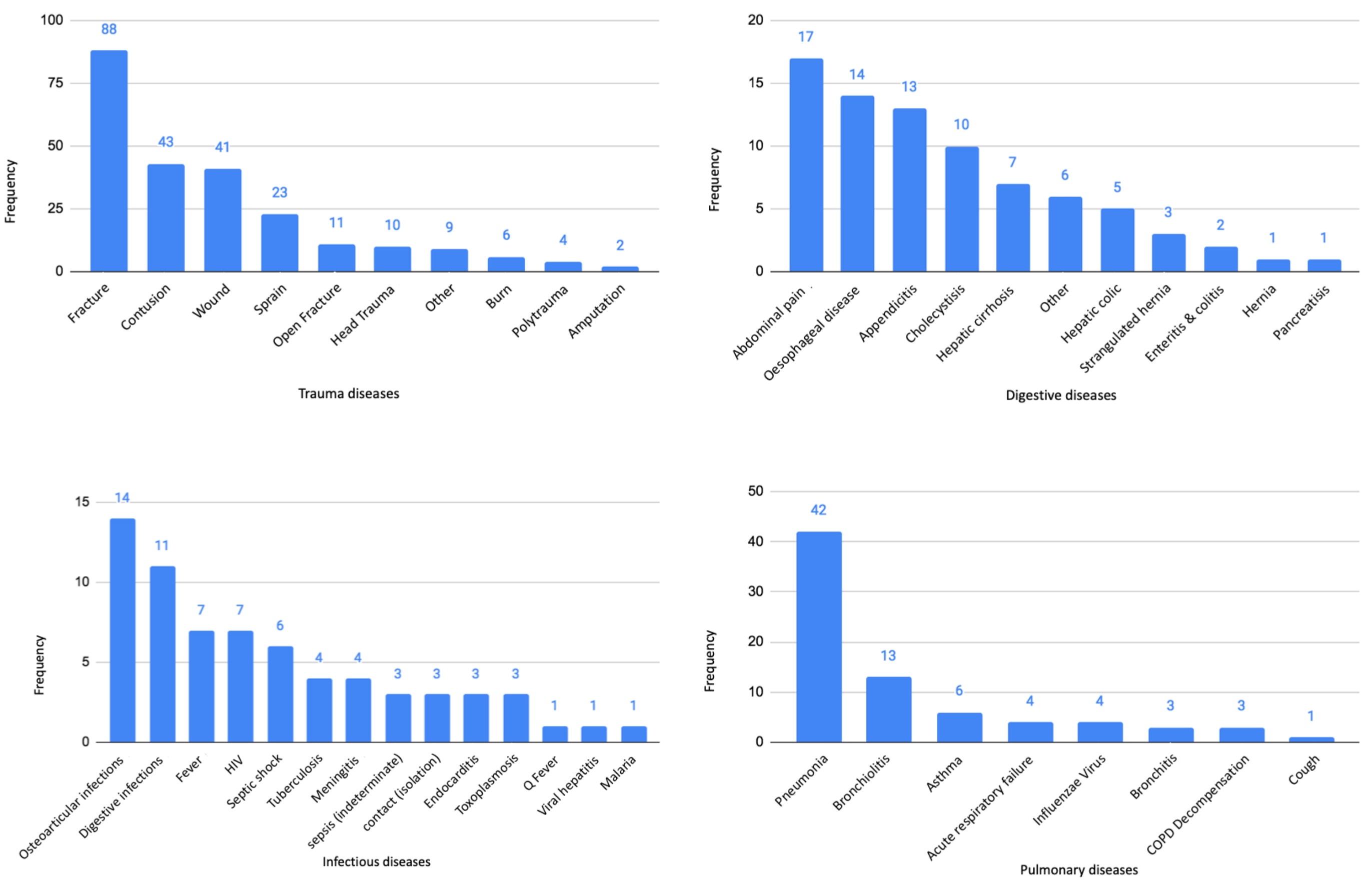

Appendix 2: Main diagnoses selected by speciality.

Appendix 3 : Clinical Classification of Emergency Patients (CCMU)

Clinical Classification of Emergency Patients	
CCMU 1	Lesion status and/or functional prognosis deemed stable.<br>No additional diagnostic or therapeutic procedures to be carried out in the emergency department.
CCMU 2	Lesion status and/or functional prognosis deemed stable.<br>Decision on additional diagnostic or therapeutic procedures to be carried out in the emergency department.
CCMU 3	Lesion status and/or functional prognosis judged likely to worsen in the emergency department without being life-threatening.
CCMU 4	Life-threatening pathological situation.<br>Management not involving immediate resuscitation.
CCMU 5	Life-threatening pathological situation.<br>Management involving immediate resuscitation.
